# Immature cystic teratoma of head of pancreas

**DOI:** 10.4103/0971-9261.43037

**Published:** 2008

**Authors:** Manoj Kela, Sangram Singh, Brijesh Lahoti

**Affiliations:** Department of Surgery and Paediatric Surgery Unit, M.G.M Medical college and M.Y.H Groups of Hospitals, Indore, Madhya Pradesh, India

**Keywords:** Pancreatic cyst, teratoma

## Abstract

Cystic pancreatic tumors are rare in children and the immature cystic teratoma of the pancreas is even rarer. A review of the world literature shows 18 documented cases involving all the age groups. The preoperative evaluation of this lesion is rather questionable, with definitive diagnosis taking place intraoperatively. We report the 19^th^ case, in a 5-month-old male child. The clinical presentation and preoperative diagnosis of this anomaly are discussed.

## INTRODUCTION

Teratomas can be divided into two subtypes: mature and immature. The mature type can be further subdivided into solid type and cystic type, hence, dermoid cyst. Derived from totipotent stem cells, they possess the ability to generate tissues from all the three germ layers: endoderm, mesoderm and ectoderm.

## CASE REPORT

**Figure 1:**
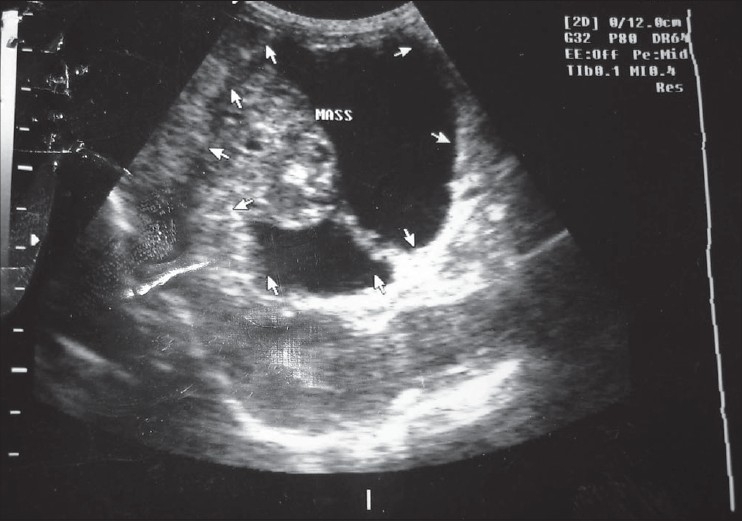
Abdominal USG showing large septate cystic mass having the possibility of a pancreatic cyst

A 5-month-old male child (weight: 7 kg.) was admitted to our hospital with a history of abdominal swelling for 1 month. His birth was normal vaginal delivery at 38 weeks of gestation and the postnatal period was unremarkable. A mobile smooth, nontender mass was palpable in periumbilical area during physical examination. Routine blood and urine biochemistry profiles were normal. The serum tumor marker AFP was normal. Abdominal ultrasonography demonstrated a large septate cystic mass (size: 8.0 × 7.0 cm) containing an echogenic solid component (size: 4.5 cm × 3.5 cm) in right lumber/hypochondriac region with a possibility of pancreatic cyst. Pancreas was not clearly observed because of the poor window [Figure 1]. CT scan and MRI could not be performed.

**Figure 2:**
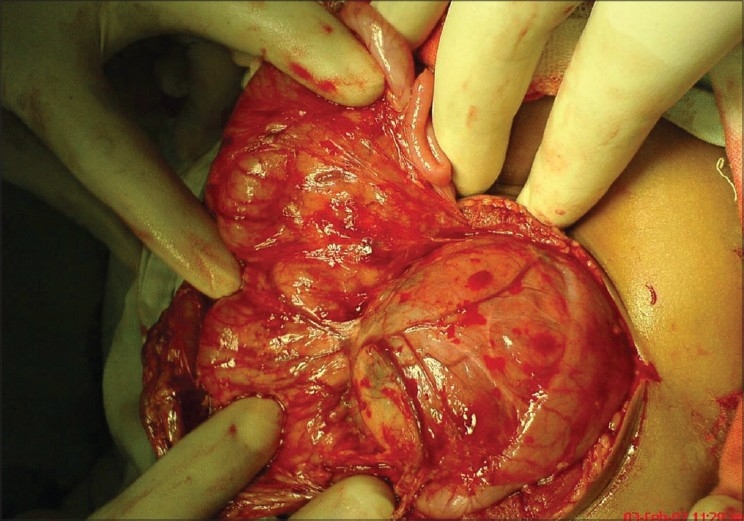
Large cystic mass arising from head of pancreas and compressing the 2^nd^ part of duodenum

**Figure 3:**
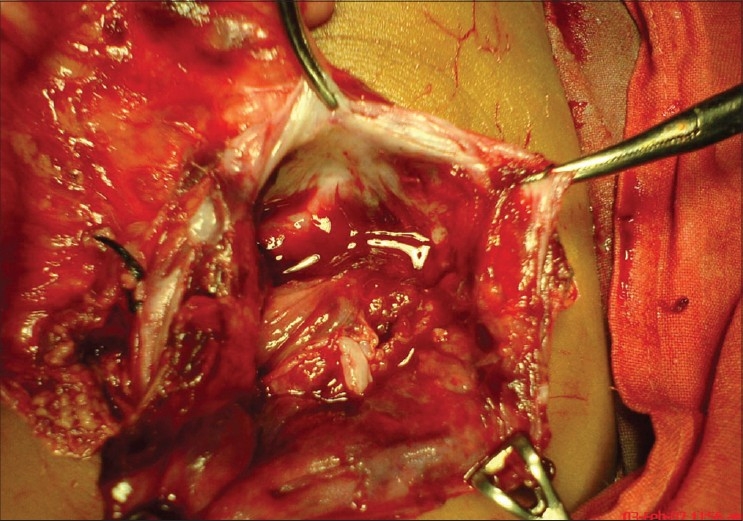
Initial deroofing of the cystic mass; well defined borders can be observed, and it contains mainly sebaceous-like matter

Surgical exploration of the patient revealed a large cystic mass at the head of the pancreas, compressing on 2nd and 3rd part of duodenum [Figure 2]. Initial deroofing showed that the mass is partially cystic in nature and contains mainly sebaceous material. Its borders were well defined and simple cystectomy was performed [Figure 3]. Histopathological findings of immature epithelial component, cartilage, fat, gastric tissue, neurovascular tissue and areas of hemorrhage and necrosis were suggestive of immature teratoma of the pancreas. Our patient had an uneventful postoperative recovery, and he was discharged on 8th postoperative day. Chemotherapy was started in view of tumor infiltration. However, soon after the first cycle of chemotherapy, the patient was lost to follow up.

## DISCUSSION

Dermoid cysts are thought to arise from the embryonic inclusion of skin at the time of neural groove closure;[1] therefore, they are typically found lying along the midline. They are most commonly located in the ovaries and have been found at several extragonadal sites, the pancreas being most rare one. Surrounding the lesion is the cyst wall that may contain adnexal tissue, sebaceous glands, lymphoid tissue and even inflammatory cells. A single layer of keratinizing squamous epithelium lies beneath this surface.[1] The inner compartment, often filled with thick, pasty, doughy sebaceous secretions, contains fully differentiated tissue(s) from one or more germ cell layers, most commonly the ectoderm.

The important distinguishing feature of teratoma is the presence of tissues derived from all three germ cell layers within a single lesion. Teratomas are classified as “mature” and “immature” teratomas, which replace the terms “benign” and “malignant” teratomas, respectively. The term immature teratoma is used for tumors containing primitive neuroectodermal, endodermal or mesodermal tissues.[2] Cells isolated from pancreas have a remarkable potential for self-renewal and multilineage differentiation.[3] Abdominal pain and backaches are the usual symptoms with or without signs of gastrointestinal obstruction. However, they may be totally asymptomatic. The symptomatology is due to tumor compression of the neighboring tissues. The differential diagnosis should include all other cystic tumors of the pancreas.[4]

Unlike dermoid cysts present elsewhere in the body, little radiographic evidence is available regarding their pancreatic location. However, by extrapolating the documented findings to the pancreas, it appears equally so that the radiological appearance of these lesions depends on the proportions of the various tissues that are affected by these lesions.[5] Ultrasound will initially define the mass as cystic, without septations and with distinct margins. The fatty component would be expected to appear as a hyperechoic components with focal areas of high-intensity signals plus acoustic shadowing, secondary to the presence of calcified tissues.[5] CT will confirm these areas of calcifications and fat and characterize the fluid as sebum, serous or complex.[5] Magnetic resonance imaging (MRI) can also be performed for further characterization. We did not proceed with MRI. However, the expected findings may include: low signal intensity on T1-weighted images areas of the fat-fluid level, if present, and distinct margins.[5]

At this point, an excisional biopsy is usually performed. However, the prospect of cytologic diagnosis should not be overlooked. In 1991, Markovsky *et al*.[6] described the findings of the first reported preoperatively diagnosed cystic teratoma by fine needle aspiration. Cytological findings included mature benign squamous cells, keratin debris and inflammatory cells and illustrated that such histological findings are inconsistent with other pancreatic disorders as pseudocysts, pancreatitis and degenerated carcinomas because of their lack of specific histological elements. Despite our failure to perform an fine needle aspiration cytology, we do believe in its selective utility in asymptomatic patients and those patients who are considered as high-risk surgical candidates. If a differential diagnosis for a cystic lesion in the pancreas has been assimilated and the radiologic evidence is inconclusive, but consistent with the above mentioned features, a fine needle aspiration for cytological analysis can confirm the diagnosis preoperatively.

Treatment is surgical extirpation, i.e., simple excision of cyst.[7] In view of paucity of the reported cases of immature teratoma, no clear cut guidelines are available in the literature with regard to the treatment and follow up of the patients with the immature teratoma, although it is well established that the total excision is the optimal treatment for the benign lesions. In general, it is a well accepted fact that the amount of neuroepithelium should guide the prognosis and treatment in such patients.[8] In our patient, however, we adopted a safer approach by giving chemotherapy (vincristine, adriamycin and cyclophosphamide). Unfortunately, the patient was lost to follow up after the first chemotherapy cycle itself, Needless to say, alpha-fetoprotein monitoring constitutes an integral part of follow up of the patients with teratoma.

Intrabdominal teratomas generally present as abdominal masses and entail good prognosis on timely treatment. Pancreatic trauma are extremely rare and are difficult to diagnose preoperatively, the diagnosis is often made in retrospect once the histology of the excised mass is studied.
